# Dementia with Lewy bodies after COVID‐19 infection with catatonia: A case report

**DOI:** 10.1002/pcn5.119

**Published:** 2023-06-28

**Authors:** Minori Tanibuchi, Shinji Ueda, Masahide Kouno, Kyohei Otani

**Affiliations:** ^1^ Department of Psychiatry Kakogawa Central City Hospital Kakogawa City, Hyogo Japan

**Keywords:** catatonia, COVID-19, dementia with Lewy bodies, dopamine-transporter scan, electroconvulsive therapy

Catatonia, a neuropsychiatric disorder, is often confused with similarly presenting diseases (delirium),^1^ resulting in fewer reports of treatable syndromes. Cases of catatonia should be recognized early and treated appropriately to avoid unnecessary morbidity and mortality. At least three of the following symptoms must be present to confirm the diagnosis: stupor, catalepsy, waxy flexibility, micturition, negativity, posturing, mannerisms, stereotypy, agitation, dysphoria, echolalia, echolocation^2^ Several cases of COVID‐19 combined with catatonia have been reported.^3^ Standard treatment for catatonia with or without COVID‐19 is benzodiazepines and electroconvulsive therapy (ECT)^4^; in addition, glutamate receptor antagonists (amantadine and memantine) are possible therapies. We describe a case of a patient with delirium and catatonia after COVID‐19 infection; catatonia appeared for the first time after the viral disease and did not respond to lorazepam. However, it improved after ECT, leading to a diagnosis of dementia with Lewy bodies (DLB).

A 68‐year‐old man, who lived alone, drank 1.5 L of beer/day, and was independent in activities of daily living (ADL) was admitted to our hospital due to COVID‐19 pneumonia and being unable to feed himself. According to his daughter, memory impairment or visual hallucinations had been not observed.

He wandered the hallways, removed his clothes, urinated in his room, and was verbally abusive and violent toward the nurses. Risperidone 0.5 mg/day was administered. His attention level and irritability fluctuated; however, he was somnolent without major disturbances, and his psychotropic medication was discontinued for 3 days after admission due to aspiration pneumonia. He occasionally said: “Dog here.” Myotonia was present in both upper extremities. Catatonia symptoms (substupor, immobility, catalepsy, and rejection) were observed. His symptoms were fluctuating and he hallucinated; the course of his illness led us to hypothesize that these were the effects of COVID‐19 infection and alcohol withdrawal delirium. The catatonia gradually worsened. Lorazepam 1 mg/day was administered, and substupor and myotonia improved (Figure 1). However, the patient's symptoms continued to fluctuate, and he remained mostly in a stupor; occasionally, he spoke spontaneously, though with hallucinatory and delusional sentences (e.g., “I'm driving now”) or repeatedly used chopsticks to bring various inedible objects to his mouth. Lorazepam was increased to 3 mg and the myotonia improved. The stupor remained unaltered and the patient was almost speechless. A head MRI showed only age‐appropriate atrophy with no other significant findings. Neurological diseases such as encephalitis were not actively suspected by neurologist's examination. The primary illness was assumed to be psychosis and withdrawal delirium, and 1 mg/day of risperidone was administered before sleep.

**Figure 1:**
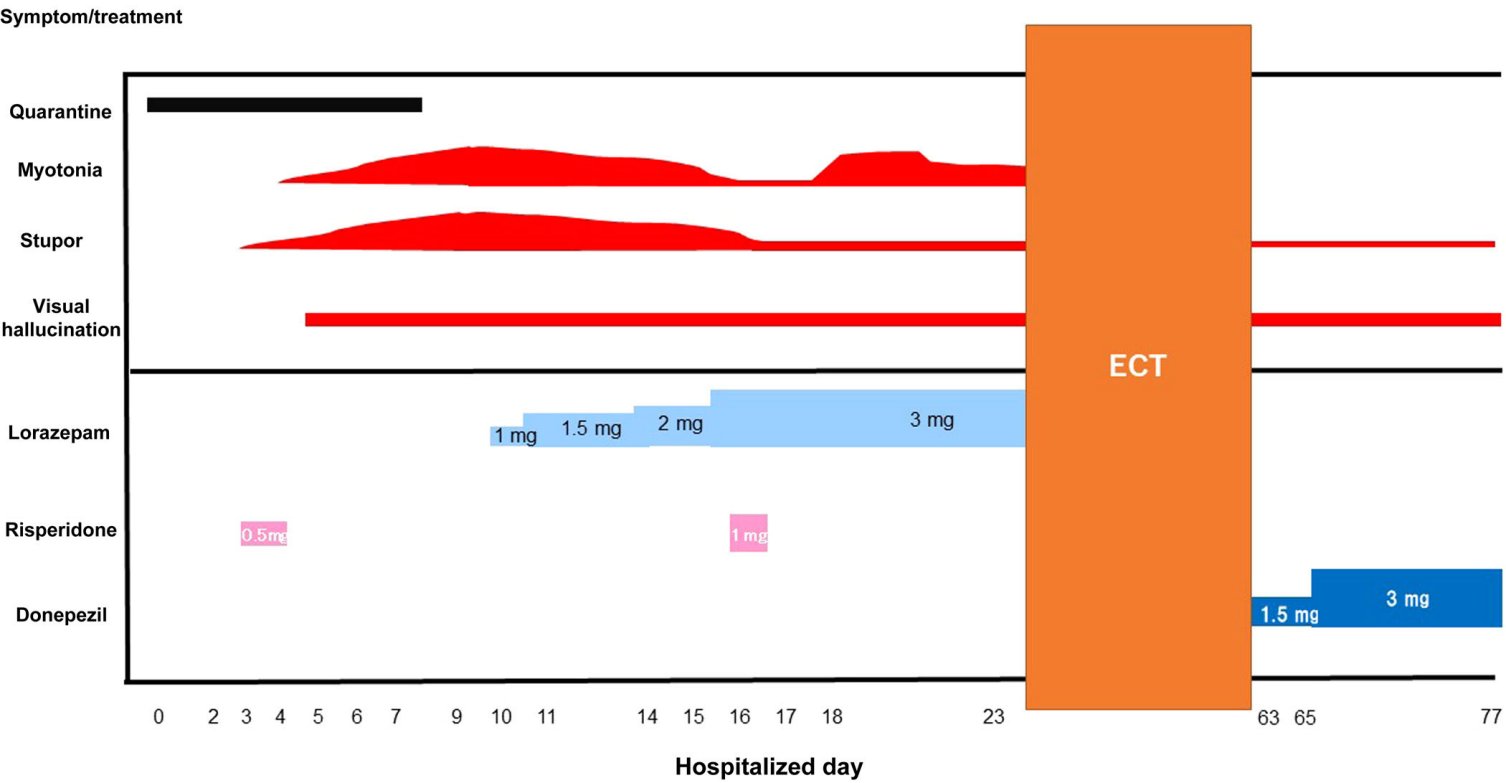
Timelines of the catatonia symptoms and treatment. Myotonia and stupor are roughly illustrated as assessed using the Bush–Francis Catatonia Rating Scale. ECT, electroconvulsive therapy.

The myotonia slightly increased, and risperidone and biperiden were stopped; however, high fever and worsening oxygenation were noted and we diagnosed aspiration pneumonia. The antipsychotic was therefore stopped due to this and worsening myotonia and substituted with lorazepam 3 mg/day, but the effects were insufficient. Malignant catatonia or malignant syndrome could not be ruled out, since he had catatonia with autonomic symptoms after starting risperidone. He was transferred to another hospital for the ECT. Six treatment sessions were performed, both myotonia and stupor improved, and the patient could move and talk freely. However, ECT was discontinued due to visual hallucinations, fluctuating alertness, cognitive decline, and irritability. Blood test results were unremarkable.

The patient returned to our hospital after 40 days for social adjustment due to ADL decline, preventing a home discharge. He scored 11/30 on the Mini Mental State Examination and showed memory impairment, disorientation, and constructive apraxia. Donepezil was started, with no improvement. He was transferred for rehabilitation.

Attention, alertness, visual hallucinations, and irritability fluctuated throughout the day and night. A forward‐leaning posture and dysphagia were prominent, whereas Parkinsonism was not. A dopamine‐transporter (DAT) scan and ^123^I‐meta‐iodobenzylguanidine imaging were performed for diagnostic purposes and showed decreased uptake, therefore DLB—not delirium—was diagnosed.

The patient's onset of catatonia led to the eventual diagnosis of DLB. Studies on α‐synucleinopathy, including DLB or Parkinson's disease (PD), regarding COVID‐19 suggest that the SARS‐CoV‐2 proteins interact with α‐synuclein, causing Lewy bodies‐like pathology in the presence of α‐synuclein overexpression.^5^ Moreover, encephalopathy has been observed in COVID‐19 pneumonia more frequently than in other pneumonias, suggesting a stronger central nervous system involvement.^6^

The clinical appearance may meet the diagnostic criteria for DLB.^7^ An increased incidence and mortality of neurodegenerative diseases, especially PD after COVID‐19 infection, has been shown,^8^ with several reports after COVID‐19.^9^ A 2.03‐fold increase in risk for Alzheimer's disease has been reported with COVID‐19 infection.^10^ This report is the first to describe a case of decreased uptake in a DAT scan and DLB diagnosis after COVID‐19 infection. Further reports are needed to confirm this association.

## AUTHOR CONTRIBUTIONS

Tanibuchi Minori, Ueda Shinji, Kouno Masahide, and Otani Kyohei were involved in the study design. Tanibuchi Minori contributed to the data analysis. Tanibuchi Minori and Otani Kyohei contributed to the data acquisition. Tanibuchi Minori drafted the initial manuscript, and Otani Kyohei revised it. All authors approved the final manuscript.

## CONFLICT OF INTEREST STATEMENT

The authors declare no conflicts of interest.

## ETHICS APPROVAL STATEMENT

Informed consent was obtained from the patient. We have obtained a release from the patient giving us permission to publish. The study was conducted in accordance with the Declaration of Helsinki. This study was not approved by the Institutional Review Board Kakogawa Central City Hospital Ethics Committee because it is a case report.

## PATIENT CONSENT STATEMENT

Written consent from the patient was obtained.

## CLINICAL TRIAL REGISTRATION

N/A.

## Data Availability

N/A.
